# A Study on the Correlation between C-Reactive Protein Concentration and Teeth with a ≥5 mm Periodontal Pocket in Chronic Periodontitis Patients

**DOI:** 10.1155/2020/8832186

**Published:** 2020-12-23

**Authors:** Hee-Yung Chang, A-Reum Kim, Sung-Hee Pi, Hyung-Keun You

**Affiliations:** Department of Periodontology, School of Dentistry, Wonkwang University, Muwangro 895, Iksan, Jeonbuk 54538, Republic of Korea

## Abstract

**Objective:**

To evaluate the relationship between CRP levels and teeth with ≥5 mm PD in chronic periodontitis patients.

**Materials and Methods:**

We evaluated 49 patients with chronic periodontitis who visited the Department of Periodontology at Wonkwang University Dental Hospital. At the first visit, high-sensitive CRP testing of venous blood samples was performed, and correlations were statistically evaluated.

**Results:**

The mean hs-CRP level of patients diagnosed with severe periodontitis was 2.0 mg/L (0.13–13.95 mg/L). Statistically, patients with a high rate of teeth diagnosed with severe periodontitis are more likely to have higher hs-CRP level.

**Conclusion:**

Within the limits of this study, the number and proportion of teeth showing ≥5 mm PD was positively correlated with CRP concentration.

## 1. Introduction

C-reactive protein (CRP) is an extremely sensitive and nonspecific acute-phase marker for inflammation that is produced by the liver in response to many forms of injury [1]. CRP contributes to host defense, and as part of the innate immune response, plasma levels of CRP rapidly rise to more than 1000-fold within hours after acute inflammatory response [1]. In clinical practice, serum CRP concentration is used to monitor infectious disease because the short half-life of CRP makes it a useful barometer for follow-up of patients with infections under antibiotic therapy [1–3].

Normal plasma CRP concentrations are on the order of 1–3 mg/L, and if the circulating CRP concentration is less than 10 mg/L, it is not clinically meaningful. However, several recent studies have reported that increased plasma CRP concentrations of 3–10 mg/L are associated with the risk of developing cardiovascular and metabolic disease [4–7]. Chronic inflammation in these diseases is thought to induce the increase in CRP concentration [1].

Chronic periodontitis is also a chronic inflammatory disease that promotes the secretion of proinflammatory cytokines such as IL-6, IL-1, and TNF-*α*. Thus, the presence of periodontitis may increase CRP levels [8]. In periodontitis, the levels of proinflammatory cytokines are also elevated, but plasma CRP levels are associated with severe infection of periodontal pathogen such as *Porphyromonas gingivalis* [9]. Therefore, several studies have been conducted on the possible correlation between periodontitis and CRP level [9–11].

Not all of these studies found an association between periodontitis and CRP; the findings differed according to the severity of the periodontitis, the degree of progression, and the target population [2]. In most previous studies, subjects were divided into several groups: healthy controls and periodontitis patients [10] or a healthy control group, moderate periodontal attachment loss group, and severe periodontal attachment loss group [9]. In another study, only differences in CRP levels between generalized periodontitis and localized periodontitis were investigated [11]. Based on this dichotomous analysis, it is difficult to explain the continuous correlation between the degree of chronic periodontitis and CRP levels.

The aim of this study was to evaluate the relationship between CRP level and the number and proportion of teeth with ≥5 mm pocket depth (PD) in moderate to severe chronic periodontitis patients.

## 2. Materials and Methods

### 2.1. Subjects

We evaluated patients diagnosed with moderate to severe chronic periodontitis who visited the Department of Periodontology at Wonkwang University Dental Hospital from December 2019 to May 2020. This study was approved by the Institutional Review Board (IRB) of Wonkwang University Dental Hospital and complied with the regulations (WKDIRB 201912-01). Individuals who had acute periodontal abscess, diabetes, cardiovascular disease, arthritis, and uncontrolled systemic disease were not included in the study. In addition, patients who received dental treatment within 3 months and smokers were excluded. Nonsmokers and those who quit smoking were included in the study. A total of 49 patients (23 men and 26 women) with available high-sensitive CRP (hs-CRP) levels through blood tests were participated, and the mean age of patients was 55.4 (38–77) years (Table 1).

### 2.2. Periodontal Examination of Periodontitis Patients

**Figure 1:**
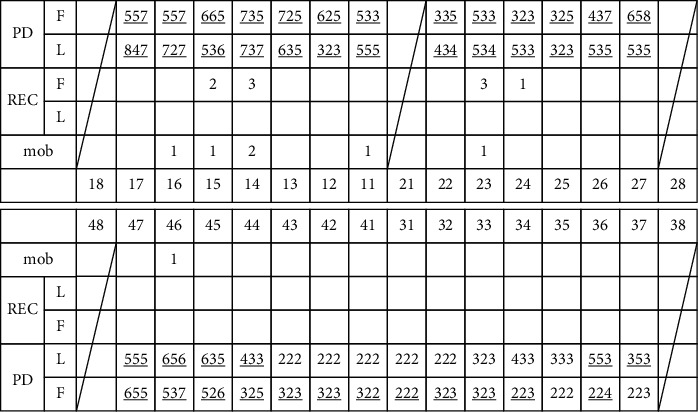
Example for periodontal examination record of one patient at first visit (mm).

At the first visit for periodontal treatment, the number of residual teeth was recorded except for third molars. Prosthetic teeth, endodontic teeth, and dental implants were included among residual teeth. PD was measured by one evaluator using a Williams probe at six sites (mesiobuccal, buccal, distobuccal, mesiolingual, lingual, and distolingual) per tooth. The number and proportion of teeth with bleeding on probing (BOP) and a ≥5 mm PD in at least one site per tooth were determined. For example, one patient had 27 residual teeth, 19 of which had ≥5 mm PD in at least 1 of the 6 sites measured, and the proportion was 70.4% (Figure 1).

### 2.3. Statistical Analysis

For statistical analysis, IBM SPSS ver. 21.0 (IBM Co., Armonk, NY, USA) was used. Pearson's correlation analysis was used to analyze the correlation between the number and proportion of teeth with *a* ≥5 mm PD and CRP concentration. The correlation between age and CRP concentration was analyzed using multiple regression analysis. Mann–Whitney *U*-test was used to determine whether there was a significant difference in CRP concentration between males and females.

## 3. Results

### 3.1. Periodontal Examination and CRP Concentration in All Patients

The mean number of residual teeth was 25.7. The mean number of teeth with ≥5 mm PD was 7.36, and the average proportion of teeth with ≥5 mm PD in the residual teeth was 29.9% in all subjects. The mean concentration of CRP was 2.0 mg/L.

### 3.2. The Relationship between the Number and Proportion of Teeth with ≥5 mm PD and CRP Concentration

**Figure 2:**
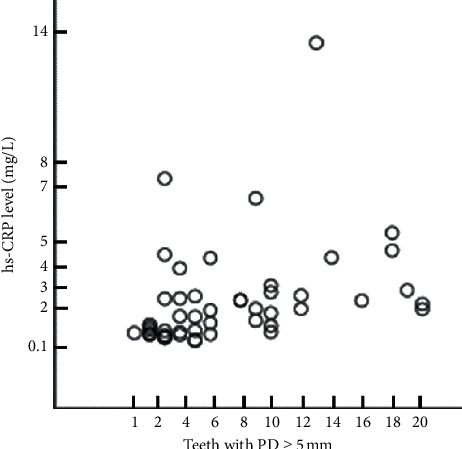
Matrix scatter plot of the number of teeth with ≥5 mm PD and CRP concentration in all subjects. The number of teeth with ≥5 mm PD was significantly associated with CRP concentration.

The correlation between the number of teeth with ≥5 mm PD and CRP concentration was analyzed using Pearson's correlation analysis for all patients participating in this study (Figure 2 and Table 2). Pearson's correlation coefficient was 0.346, and the number of teeth with ≥5 mm PD was significantly associated with CRP concentration (*p* < 0.05).

**Figure 3:**
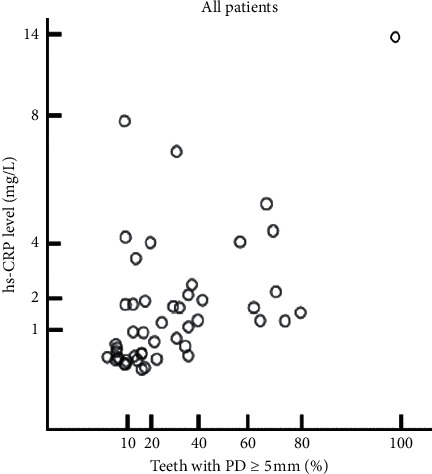
Matrix scatter plot of the proportion of teeth with ≥5 mm PD and CRP concentration in all subjects. The proportion of teeth with ≥5 mm PD was significantly associated with CRP concentration.

The correlation between the proportion of teeth with ≥5 mm PD and CRP concentration was analyzed using Pearson's correlation analysis for all patients participating in this study (Figure 3 and Table 2). Pearson's correlation coefficient was 0.51, and the proportion of teeth with ≥5 mm PD was significantly associated with CRP concentration (*p* < 0.05).

**Figure 4:**
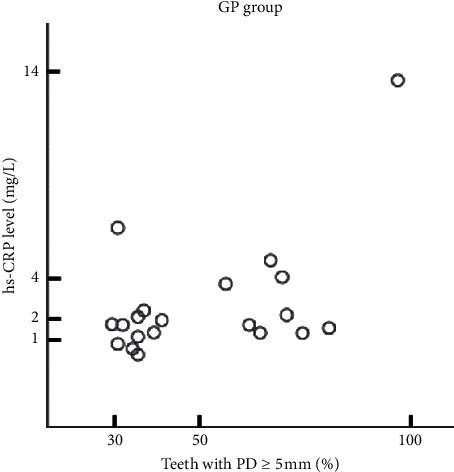
Matrix scatter plot of the proportion of teeth with ≥5 mm PD and CRP concentration in the CPG group. The proportion of teeth with ≥5 mm PD was significantly associated with CRP concentration in the CPG group.

**Figure 5:**
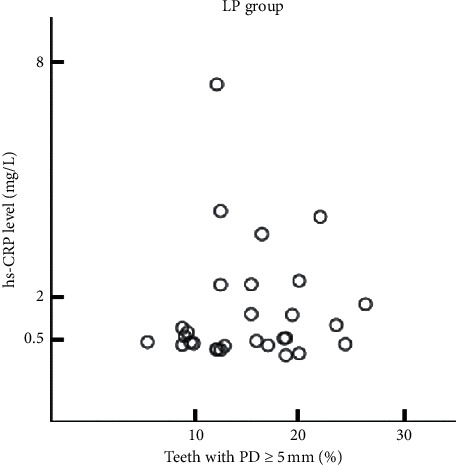
Matrix scatter plot of the proportion of teeth with ≥5 mm PD and CRP concentration in the CPL group. The proportion of teeth with ≥5 mm PD was not significantly associated with CRP concentration in the CPL group.

In addition, all subjects were divided into two groups: those with a proportion of ≥5 mm PD teeth of 30 % or less (chronic periodontitis-localized group, CPL group) and 30 % or more of total residual teeth (chronic periodontitis-generalized group, CPG group). We analyzed the relationship between the proportion of teeth with ≥5 mm PD and CRP concentration in the CPG and the CPL groups. Pearson's correlation coefficient was 0.517 in the CPG group with a *p* value of 0.019, which showed a statistical significance (Figure 4 and Table 3). In the CPL group, Pearson's correlation coefficient was 0.060, and the *p* value was 0.758, with no statistically significant correlation (Figure 5, Table 3).

### 3.3. Relationship between Age, Gender, and CRP Concentration

We analyzed the correlation between age and CRP concentration among the patients participated in this study using multiple regression analysis (*p*=0.452; Table 4). There was no statistically significant correlation.

On regression analysis, gender did not affect CRP concentration (*p*=0.881). The Mann–Whitney *U*-test was used to determine whether there was a significant difference in CRP levels between men and women. The *p* value was 0.180, and there was no statistically significant difference (Table 5).

## 4. Discussion

Within the limits of this study, in patients with chronic periodontitis, CRP concentration continuously increased as the number of residual teeth increased.

Many studies have reported an increased CRP level in the presence of periodontitis. These studies showed that greater periodontitis severity and wider distribution are associated with higher CRP concentration. Filho et al. reported that the mean CRP concentration (2.6 ± 2.6 mg/L) in patients with chronic periodontitis was higher than that in patients without chronic periodontitis (1.78 ± 2.7 mg/L) [10]. CRP concentration tends to be higher in elderly, smoking, and diabetic patients, and chronic periodontitis was also associated with an increase in CRP levels when these variables are controlled. Noack et al. reported that CRP levels were significantly higher in patients with 3 mm or more attachment loss (4.06 ± 5.55 mg/L) than among those with 2-3 mm attachment loss (2.78 ± 5.14 mg/L) and no attachment loss (1.70 ± 1.91 mg/L). In patients with 3 mm or more attachment loss, the proportion of patients with CRP levels above 3 mg/L (38.0 %) was significantly higher than among those with 2-3 mm attachment loss (23.7 %) and no attachment loss (16.9 %) [9].

In patients with aggressive periodontitis, the mean CRP level is higher in generalized aggressive periodontitis (3.72 mg/L) than localized aggressive periodontitis (2.57 mg/L) and controls (1.5 4 mg/L) [11].

In previous research, the correlation between periodontal disease and CRP level was analyzed in a dichotomous manner. But in this study, it was further subdivided and analyzed according to the number and proportion of teeth affected by periodontitis. In this study, the mean number of residual teeth was 25.7, and the mean number of teeth with ≥5 mm PD was 7.36. The average proportion of teeth with ≥5 mm PD was 29.9 %. The mean CRP concentration was 2.0 mg/L. There was a continuous correlation between the number and proportion of teeth with ≥5 mm PD and the CRP concentration.

High levels of inflammatory cytokines such as IL-1, IL-6, and TNF-*α* are seen in the periodontal patients. When periodontal tissue is destroyed, these cytokines are secreted and affect the whole body through blood circulation. As the number and proportion of infected teeth increase, the secretion of cytokines increases, and therefore, CRP also increases continuously [12].

In this study, the highest CRP concentration (13.95 mg/L) was observed in a patient with 13 residual teeth. 9 of 13 teeth showed attachment loss to the apex on radiographs, and 4 teeth showed about 10 mm of PD. Even if several teeth were extracted with severe periodontitis, the residual teeth may be at risk for severe periodontitis. Therefore, there is likely to be a significant correlation between CRP concentration and the proportion of residual teeth with ≥5 mm PD.

In the study, the correlation between CRP concentration and the proportion of teeth with ≥5 mm PD in the CPG and CPL groups was analyzed. The CPG group showed a statistically significant positive correlation, while the CPL group had no significant correlation. The large distribution of teeth with deep periodontal pockets in the CPG group means that the degree of inflammation is more severe than CPL. CRP is an acute reactant that increases or decreases in response to inflammation or tissue damage, and CRP levels are more significantly relevant in CPG where the degree of inflammation is severe. However, more research is needed on this.

It has been reported that aging tends to increase CRP concentration because the elderly have more fat accumulation and more inflammatory diseases [10]. However, age did not correlate with CRP concentration in this study. As the increase in age was not a direct cause of periodontal disease [13], it was considered that the increase in the age of periodontal disease does not increase the CRP concentration together.

In this study, gender differences between men and women did not affect CRP levels. Noack et al. reported no correlation between sex and CRP concentration [9], while Woloshin and Schwartz reported that women had higher CRP levels than men [14]. CRP levels may increase with pregnancy, menopause, or estrogen in women [15]. Men are reported to have more severe periodontal destruction than women [16]. Further studies are needed to compare the CRP levels of men and women with periodontal disease.

One limitation of this study is that the total number of subjects was small, as we excluded patients with systemic diseases that might affect CRP levels such as cardiovascular disease, diabetes, arthritis, and uncontrolled systemic disease. Another limitation is that patients were presumed to be systemically healthy based on self-reporting, and included subjects could have had mild disease that may have affected CRP levels.

In future studies, detailed evaluation will be needed to determine whether CRP concentration is influenced by various periodontal conditions such as furcation involvement, floating state of teeth, and the area of the periodontal pocket according to the tooth location.

## 5. Conclusion

The purpose of this study was to evaluate the relationship between CRP levels and the number and proportion of teeth that showed with ≥5 mm PD in chronic periodontitis. Within the limits of this study, the number and proportion of teeth with ≥5 mm PD were positively correlated with CRP concentration.

## Figures and Tables

**Table 1:** Subject characteristics.

Subject	Gender	Age	Number of residual teeth	Number of teeth with ≥5 mm PD	Proportion of teeth with ≥5 mm PD (%)	hs-CRP level (mg/L)
1	F	57	28	12	42.9	2.23
2	F	53	28	3	10.7	0.30
3	F	60	27	10	37.0	2.39
4	F	54	28	5	17.9	0.13
5	M	46	26	3	11.5	0.39
6	F	46	28	3	10.7	0.29
7	F	52	26	5	19.2	0.18
8	M	52	26	5	19.2	2.20
9	F	58	28	2	7.1	0.42
10	M	50	25	18	72.0	4.33
11	M	56	28	10	35.7	0.83
12	M	46	24	2	8.3	0.46
13	M	64	28	5	17.9	0.61
14	F	66	28	9	32.1	6.74
15	M	63	28	1	3.6	0.50
16	M	68	26	8	30.8	2.03
17	M	56	28	9	32.1	1.07
18	M	49	27	3	11.1	4.14
19	F	45	25	6	24.0	0.44
20	F	48	24	20	83.3	1.85
21	M	54	27	5	18.5	1.25
22	F	58	27	3	11.1	0.28
23	F	56	24	14	58.3	4.00
24	M	49	26	10	38.5	2.70
25	F	45	28	2	7.1	0.89
26	F	62	26	4	15.4	3.50
27	M	74	17	3	17.6	0.60
28	M	64	22	9	40.9	1.62
29	F	52	13	13	100.0	13.95
30	M	58	27	2	7.4	0.66
31	M	58	25	16	64.0	2.00
32	M	42	27	10	37.0	0.54
33	F	56	26	18	69.2	5.15
34	F	47	28	4	14.3	2.10
35	F	62	26	19	73.1	2.48
36	M	54	28	6	21.4	3.98
37	M	60	24	8	33.3	2.00
38	M	49	28	4	14.3	1.27
39	M	58	18	12	66.7	1.61
40	F	46	27	10	37.0	1.42
41	F	60	25	4	16.0	0.41
42	M	38	28	3	10.7	7.66
43	F	58	23	6	26.1	1.55
44	F	76	27	4	14.8	0.53
45	F	77	27	3	11.1	2.09
46	F	45	25	2	8.0	0.49
47	F	67	26	20	76.9	1.60
48	M	62	26	6	23.1	0.97
49	F	38	26	2	7.7	0.77
Mean		55.4	25.7	7.36	29.9	2.0

**Table 2:** Association of the number and proportion of teeth with ≥5 mm PD and CRP concentration.

	CRP concentration	
	Pearson's correlation coefficient (*r*)	*p* value
Number of teeth showing with ≥5 mm PD	0.346	0.015^*∗*^
Proportion of teeth showing with ≥5 mm PD	0.510	0.001^*∗*^

Pearson's correlation coefficient. ^*∗*^Significant difference (*p* < 0.05).

**Table 3:** Association of the proportion of teeth with ≥5 mm PD with the CRP concentration in the CPG and CPL groups.

	CRP concentration	
	Pearson's correlation coefficient (*r*)	*p* value
CPG	0.517	0.019^*∗*^
CPL	0.060	0.758

Pearson's correlation coefficient. ^∗^Significant difference (*p* < 0.05).

**Table 4:** Association of age with CRP concentration.

	CRP concentration	
Age	*β*	−0.084
	*p* value	0.452

	*F* = 12.221, *p*=0.000, *R*^2^ = 0.449, adj*R*^2^ = 0.412, DW = 1.85	

Multiple regression analysis.

**Table 5:** Association of gender with CRP concentration.

	CRP concentration		
Gender	*p* value		0.881
	Men	Women	*p* value
	2.08 ± 1.83	1.98 ± 2.86	0.180

Pearson's correlation coefficient, Mann–Whitney *U*-test (mean ± SD (mg/L)).

## Data Availability

The data are not available for public access because of patient privacy concern.
